# Supplementary material for: Comparison of the efficiency of Deebag and jute made bag for faecal sludge management and wastewater treatment
Source: PLoS One. 2020 Nov 11;15(11):e0241046. doi: 10.1371/journal.pone.0241046 (PMC7657504; doi:10.1371/journal.pone.0241046)
Supplement: Attachment — Submitted filename: Response to Reviewers.doc [file pone.0241046.s011.doc]

**Response to the Comments of Reviewer#1**

1) Please, use the same word for vacutug. In the lines 77, 89 and 91 (related to clean version) “vacutug” is used; in the lines 83 and 85 “vacutag” is adopted.

**Response:** “Vacutug” is the right word. I have replaced vacutag with vacutug in the lines 83 and 85 of the clean version. It has been also corrected in the manuscript with track changes.

2) Replace PO43+ with PO43- (e.g. in the line 192 of clean version). Please, check the whole manuscript. Moreover, I suggest to write BOD5 with “5” in subscript mode.

**Response:** I have replaced PO_4_^3+^ with PO_4_^3-^ in the clean version of manuscript. I have also write BOD5 with BOD_5_ that is “5” in subscript mode in the clean version of the manuscript. Both changes have also been done in the manuscript with track changes.

3) In the manuscript with track of change mode, the Figure 3 does not show the removal percentage of EC. In the clean version the Figure 3 is corrected.

**Response:** This was my mistake and I am extremely sorry for that. I have made change the Figure 3 with the removal percentage of EC in the manuscript with track changes.

4) Line 358 (of clean version): I suggest to replace “According to Rose and Strande et al.,” with “According to Rose et al. and Strande et al.”.

**Response:** In the line 358 of clean version of the manuscript, “According to Rose and Strande et al.,” has been replaced with “According to Rose et al. and Strande et al.”. This change has also been done in the manuscript with track changes.

5) Line 385 (of clean version): “... for other analyzed parameters”. I suggest to specify the other parameters.

**Response:** The other analyzed parameters have been specified as COD, EC, Cl^-^, PO_4_^3-^ and TSS in the line 385 of clean version of the manuscript. Same changes have been also done in the manuscript with track changes.
